# Role of Scaffolds, Subchondral, Intra-Articular Injections of Fresh Autologous Bone Marrow Concentrate Regenerative Cells in Treating Human Knee Cartilage Lesions: Different Approaches and Different Results

**DOI:** 10.3390/ijms22083844

**Published:** 2021-04-08

**Authors:** Jacques Hernigou, Pascale Vertongen, Joanne Rasschaert, Philippe Hernigou

**Affiliations:** 1 Department of Orthopedic Surgery, EpiCURA Hospital, 7331 Baudour, Belgium; Jacques.hernigou@gmail.com; 2 Laboratory of Bone and Metabolic Biochemistry, Faculty of Medecine, Université Libre de Bruxelles, 1070 Brussels, Belgium; pascale.vertongen@ulb.ac.be (P.V.); joanne.rasschaert@ulb.ac.be (J.R.); 3 Department of Orthopaedic Surgery, Faculty of Medicine, UPEC (University Paris-Est, Créteil), 94000 Créteil, France

**Keywords:** bone marrow concentrate, bone marrow aspirate, mesenchymal stem cells, osteoarthritis, cartilage, regeneration, intra-articular injection, MSCs subchondral injection, MSCs loaded scaffolds

## Abstract

The value of bone marrow aspirate concentrates for treatment of human knee cartilage lesions is unclear. Most of the studies were performed with intra-articular injections. However, subchondral bone plays an important role in the progression of osteoarthritis. We investigated by a literature review whether joint, subchondral bone, or/and scaffolds implantation of fresh autologous bone marrow aspirate concentrated (BMAC) containing mesenchymal stem cells (MSCs) would improve osteoarthritis (OA). There is in vivo evidence that suggests that all these different approaches (intra-articular injections, subchondral implantation, scaffolds loaded with BMAC) can improve the patient. This review analyzes the evidence for each different approach to treat OA. We found that the use of intra-articular injections resulted in a significant relief of pain symptoms in the short term and was maintained in 12 months. However, the clinical trials indicate that the application of autologous bone marrow concentrates in combination with scaffolds or in injection in the subchondral bone was superior to intra-articular injection for long-term results. The tendency of MSCs to differentiate into fibrocartilage affecting the outcome was a common issue faced by all the studies when biopsies were performed, except for scaffolds implantation in which some hyaline cartilage was found. The review suggests also that both implantation of subchondral BMAC and scaffolds loaded with BMAC could reduce the need for further surgery.

## 1. Introduction

Mesenchymal stromal cells (MSCs) are promising alternatives for the treatment of osteoarthritis (OA) due to their tissue repair capacity and their secretion of bioactive factors [1,2]. MSCs can be obtained from bone marrow, synovial membrane and adipose tissue [3]. Bone marrow MSCs (BM-MSCs) were the first identified, and bone marrow represents a common source of MSCs [4]. These cells can be applied after culture expansion or injected as bone marrow concentrate (BMC). Considering regulations on cell expansion, the use of cultured BM-MSCs in Europe and in the USA [5,6,7,8] is limited. Conversely, bone marrow aspirate concentrate (BMAC), considered as a minimal cell manipulation, allows one to obtain a product that can be used in clinical practice to treat degenerative cartilage lesions in a one-step treatment.

The therapeutic potential of BMAC (with or without scaffold) has been first tested for OA treatment and full cartilage defect in five animal studies with or without a scaffold. These comparative studies [9,10,11,12,13] investigated the results of BMAC, against placebo (saline), platelet-rich plasma (PRP), hyaluronic acid (HA), and cultured BMSCs in the knee OA of rabbit, sheep, and goat. The evaluation of the articular cartilage showed better macroscopic results in animals treated with BMAC as compared with control groups. The histological results were also better in the BMAC group. Immunohistochemical analyses [9] demonstrated an effect on regulation of type I collagen and of TNF-α after BMAC treatment as an increase of proteoglycan concentration in the BMAC groups. Gene expression analyses showed decreased expression of MMP- 13 and increased expression of Col2A1 and Aggrecan in all cell groups as compared with saline [10]. 

Therefore, BMAC in a one-step treatment has also been used in clinical studies as an approach to treat degenerative joints of the knee. Treatments consist of the administration of bone marrow concentrates [14] in the joint or in a scaffold impacted on the subchondral bone [15] or directly in the subchondral bone. The last application [16] has been designed to target the subchondral bone which is frequently affected by the OA processes. The advent of MRI during the two last decades has underlined the role of the subchondral bone in OA pathology [17]. Bone marrow lesions (as synovitis) are now used as a marker for OA because as they may reverse with OA treatment. Therefore, there is at this moment three different ways to administer BMAC for the treatment of an osteoarthritic knee: intra-articular BMAC injections without loading cells in the subchondral bone, i.e., just targeting cartilage and synovitis; BMAC subchondral injections with the idea that the subchondral bone is abnormal (with a decrease of MSCs) and could be improved and repaired by injecting cells; surgical implantation of scaffolds supercharged with BMAC, with the idea that the cartilage and the pathological subchondral bone cannot be repaired and should be changed with a scaffold loaded with cells.

The purposes of this narrative review are to summarize the rationale and the results of each of these techniques of autologous therapies with BMAC, and to discuss how each technique may intervene in the repair of human OA disease.

## 2. Intra-Articular BMAC Injections Targeting Cartilage and Synovitis

### 2.1. Destructions of the Cartilage and Occurrence of Inflammatory Synoviopathy

Hyaline cartilage is a specialized connective tissue (nonvascularized, non-innervated). It protects bone from mechanical stresses, absorbs shocks and allows a frictionless surface for movements [18]. Elasticity and resistance are properties which allow the cartilage to support a force of up to 6 times the body’s weight with a thickness of only 4 mm. A single type of cell, the chondrocyte (representing approximately 1% of the cartilage’s volume), constitutes this tissue. Cells are dispersed in an extracellular matrix composed of 70–80% water, a network of Type II (90 to 95%) collagen fibers surrounding proteoglycans (agrecans), being grouped in high molecular weight aggregates. The regeneration of the matrix [18] is slow—200 years for collagen in adults. Collagen synthesis decreases with age, causing deterioration faster than it is replaced. Irreversible traumatic lesions of cartilage result in disorganization of collagen network and modify the chondrocytes’ capacities to support the mechanical stresses. The progression towards osteoarthritis is frequent, especially in weight bearing regions. 

Normally, it is believed that, following cartilage lesions, synovitis occurs. This may be related to the damaged cartilage’s release of breakdown products such as hyaluronan, or cytokines activated by damage of other proteins. However, in some circumstances, synovitis could occur before cartilage lesion [19]; MRI after a marathon race shows joint effusion, but without cartilage damage [20]. As the two main clinical symptoms of OA (pain and joint stiffness) are related to synovial inflammation, a treatment targeting both cartilage and synovitis by intra-articular injection appears logical. Furthermore, some factors, such as IL-1, IL-6 and TNF produced by the synovial membrane after a macrophage activation, diffuse via the synovial fluid into the cartilage. These factors can lead to apoptosis of chondrocytes [20]. A pannus (infiltrating connective tissue) also may occur in OA and induce bone and cartilage damage. Osteoarthritis-induced synovitis is histologically a proliferation and a hyperplasia of cells with inflammatory cell infiltration and neoangiogenesis [21]. In knee OA, synovitis is predominantly located in the suprapatellar region [22] with an easy access for intra-articular injection. 

### 2.2. The Rational of Using BMAC for Intra-Articular Pathology

**Figure 1:**
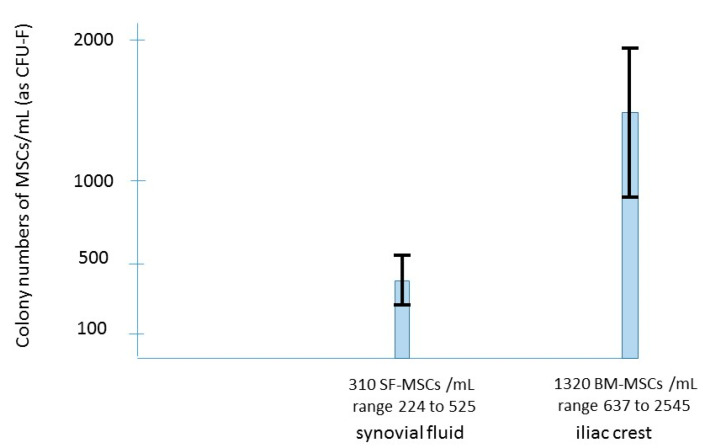
Comparison among synovial fluid MSCs and bone marrow iliac crest MSCs from osteoarthritis patients.

**Figure 2:**
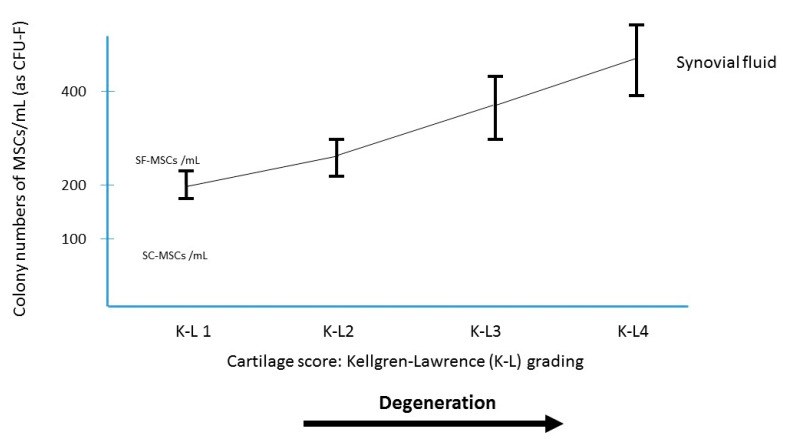
MSCs in synovial fluid derived from osteoarthritis patients. Relationship between the osteoarthritis grading and the colony number of synovial fluid MSCs per synovial fluid volume (mL).

It is now admitted that the joint cavity may be considered as a reservoir of MSCs which might play for cartilage a role for joint homeostasis and for repair [23,24]. The presence of MSCs in the synovial fluid of knees with primary OA was first reported by Jones [23] and is numerically increased even in early osteoarthritis [24]. These MSCs are probably mobilized from the synovium (through the synovial fluid) towards the degenerative cartilage. Despite this increase, the number of MSCs in the synovial fluid (SF) of patients with knee OA remains lower than the number of MSCs in the iliac crest of the same patient (Figure 1). Sekiya [24], Davies [25] and Hernigou [26] evaluated the number of MSCs in the joint fluid of OA patients; the SF-MSCs (CFU-F per ml of synovial fluid) were low (average in the three studies 310 SF-MSCs/mL; range 65 to 525) compared to the iliac crest BM-MSC-aspirate crude number (1320 MSCs/mL; range 637 to 2545) of the same patients (before concentration). Therefore, a treatment increasing their number with intra-articular injection of BMAC could be a hypothesis to inhibit the progression of osteoarthritis. Comparing the Kellgren–Lawrence (K-L) Grades [27] of osteoarthritis groups is interesting: MSCs increased in synovial fluid (Figure 2) according to the radiological severity graded by Kellgren–Lawrence classification of all the three studies [24,25,26]. 

### 2.3. Results of Clinical Studies Using Intra-Articular BMAC

Autologous bone-marrow-derived stem cells were reported by several prospective or retrospective studies [28,29,30] demonstrating improvements of symptoms in knee OA. More interesting, in 5 randomized studies (Table 1), BMAC was compared to saline, hyaluronic acid, exercise, PRP, and injection in the subchondral bone.

- Autologous bone marrow-derived stem cells vs. saline: In a prospective, single-blind, placebo-controlled trial, 25 patients with bilateral knee pain from bilateral osteoarthritis were randomized by Shapiro [31] to receive BMAC into one knee and saline placebo into the other. Bone marrow was aspirated from the iliac crests and concentrated. BMAC or saline were injected into each arthritic knee thereby utilizing each patient as his own control. VAS pain scores in both knees decreased significantly from baseline at 6 months. However, pain relief, although dramatic, did not differ significantly between treated knees.
- Autologous bone-marrow-derived stem cells vs. exercise: In this study, Centeno [32] performed an injection of BMAC versus exercise therapy in 48 patients. Patients who received BMAC injection had better Knee Society Score (KSS) compared to control group after 3 months. Better results were found for BMAC injections against exercise therapy.
- Autologous bone-marrow-derived stem cells vs. hyaluronic acid: Goncars et al. [33] investigated the effect of a single injection with autologous bone-marrow-derived mononuclear cells versus three injections of HA performed 1 week apart. After 12 months, clinical scores improved significantly in both groups. KSS (Knee Society Score) improved in both groups as well and there was no statistically significant difference between these 2 groups.
- Autologous bone-marrow-derived stem cells vs. PRP: Anz [34] evaluated a total of 90 participants with symptomatic knees OA (Kellgren-Lawrence grades 1–3). Patients were randomized into 2 study groups: PRP and BMAC. All clinical scores for both the PRP and BMAC groups significantly improved from baseline to 1 month after the injection. These improvements were sustained for 12 months after the injection, with no difference between PRP and BMAC at any time point.
- Autologous bone-marrow-derived stem cells injected intra-articularly versus subchondral bone injection: in a randomized controlled clinical trial performed between 2000 and 2005 in 120 knees of 60 patients with a similar osteoarthritis grade, Hernigou [26] injected the same amount of BMAC in the joint of one knee and in the subchondral bone of the contralateral knee. Concerning the side with an intra-articular injection, 20 mL containing average 5727 MSCs/mL (range 2740 to 7540) were injected in the joint; the relative increased, compared to baseline number in the joint before injections) ranged from 5-fold to 30-fold, depending of the OA severity and age. Effusions were seen for several (<15) days after the procedure in both knees; these were anticipated findings, and probably these initial effusions were likely to be a residual of the 20-mL infiltration performed in each knee. The mean overall changes in knee scores (improved until 2-year follow up) from 52 points ±15 to 64 points ±21. However, with radiographs and MRI, no improvement was observed for joint space, bone marrow lesions and synovitis.

**Table 1:** Clinical randomized studies with BMAC intra-articular injections.

Author	Study Design	Comparison	Nb of Knees	Age	Follow-Up	MRI
Shapiro [31]	blinded RCT	BMAC vs. saline	25/25	60(42–68)	1 year	No
Goncars [33]	Un-blinded RCT	BMAC vs. HA	28/28	53 ± 15	1 year	No
Centeno [32]	Un-blinded RCT	BMAC vs. exercise	26/22	54 ± 9/57 ± 8	2 years	No
Anz [34]	Un-blinded RCT	BMAB vs. PRP	45/41	56 ± 11/52 ± 12	1 year	No
Hernigou [26]	Blinded RCT	BMAC	60/60	76(62–87)	15 years	Yes
Intra-articular vs. subchondral						

In summary, for intra-articular injections with BMAC, pain scores improved, but no comparative study reported a superiority of BMAC against another injective treatment even for placebo, and no MRI improvement was observed on the disease. 

### 2.4. Proposed Mechanisms of Action of BMAC Injected in the Joint

Although intra-articular injection was able to reduce pain during the first 12 months, it was not able to reduce synovitis or to decrease BMLs in the subchondral bone, and the pain improvement was not sustained in the long term; we can only speculate on the early improvement and the late failure. Studies have reported that MSCs can inhibit the NFκB pathway and decrease the inflammatory response of synovium and articular cartilage [35]. In addition, MSCs may reduce pain by targeting the cannabinoid receptors [36] of synovial cells. This may explain the early benefit answer after their injection. However, their repair capacity might be limited due to interactions with SF hyaluronan that limit their adhesion to cartilage; additionally, the synovium, cartilage, and other joint structures share a common progenitor that is distinct from that of bone tissue [24], suggesting that the synovium harbors a joint-tissue-specific stem-cell population, which could explain less effective results with bone marrow MSCs injected in the joint. 

## 3. Subchondral Injections BMAC Without Scaffold

### 3.1. The Subchondral Bone Damage in OA

The notion that cartilage degeneration is the primary mechanism of OA is now discussed, in view of changes present in the subchondral bone which suggest that this tissue may play a role in the disease. The severity of subchondral bone damage is higher than cartilage changes when OA is beginning in animals and humans [37,38]. Frequently observed by MRI, bone marrow lesions (BMLs) are probably a consequence of remodeling in the subchondral bone and rarely spontaneously regress [39]. Associated with these bone marrow changes, abnormal biochemical parameters (including cytokines, growth factors, and inflammatory mediators) are observed in OA subchondral bone. The levels of many markers (alkaline phosphatase, osteocalcin, collagen type l, TGF-J3, PGE_2_, LTB_4_, IL-6, IGF-1, urokinase, cathepsin K and metalloproteases) have all been found abnormal in subchondral osteoblasts [40,41,42,43] in patients with OA. Some of these abnormalities could be linked to a pathologic process in bone marrow. Aspden et al. [44] introduced the idea that OA is a systemic and metabolic disease related to the dedifferentiation of mesenchymal stem cells in bone marrow. 

### 3.2. The Rational of Using BMAC for Subchondral Pathology

**Figure 3:**
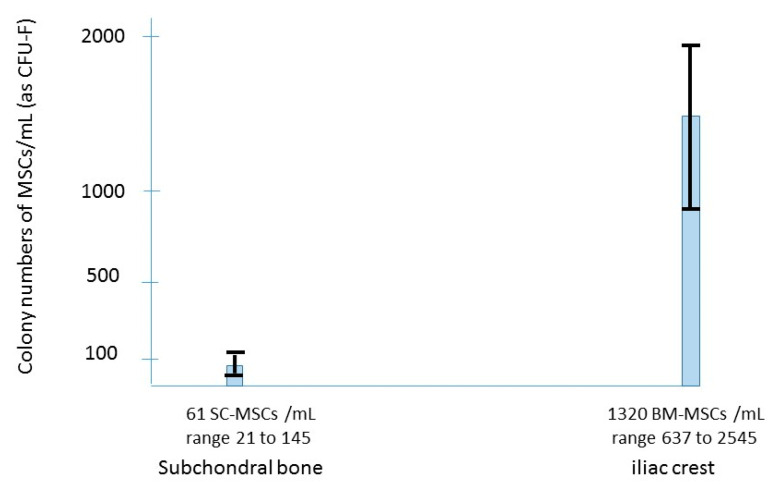
Comparison among subchondral bone marrow MSCs and bone marrow iliac crest MSCs from osteoarthritis patients.

**Figure 4:**
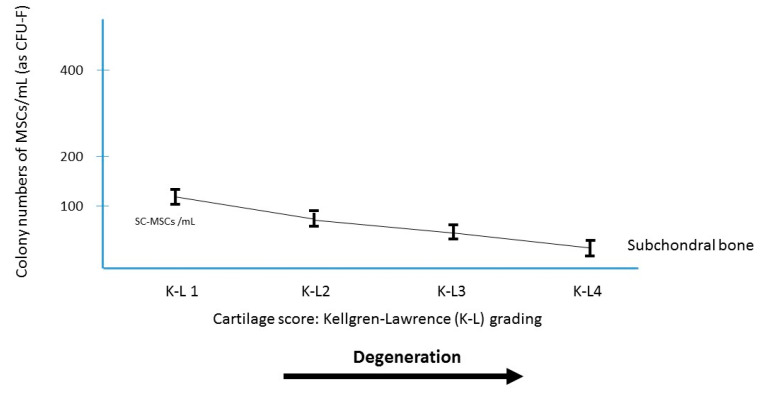
MSCs in subchondral bone derived from osteoarthritis patients. Relationship between the osteoarthritis grading and the colony number of subchondral bone marrow aspirate (mL).

The number of MSCs present in the subchondral bone of the knee decreases with age [23,24,25,26], suggesting that such MSC deficit could prime the degenerative process in elderly patients. In terms of number of MSCs per ml of bone marrow aspirate in the subchondral bone of OA knees, this number was analyzed by several authors [23,24,25,26]. The subchondral SC-MSCs (CFU-F per ml of bone marrow aspirate) were few in OA knees (Figure 3), 65 MSCs/mL (range 33 to 145) for the femur, and 53 MSCs/mL (range 21 to 119) for the tibia, lower than the SF-MSCs number in the joint fluid of the same knees (Figure 1), and very few as compared to the iliac crest BM-MSC-aspirate crude number of the same patients. This decrease in MSCs in the subchondral bone of the joint occurs not only with age but also with OA (Figure 4). Thus, there could be some advantage to injecting MSCs in the subchondral bone of OA patients where they can provide many bioactive mediators which have been shown to exert positive effects on joint tissues. With assumption that in OA the abnormality of the subchondral bone is related to a dysfunction of mesenchymal stem cells (low number and/or abnormal function), this deficiency might be corrected by transplantation of MSCs from the iliac crest of the patient. 

### 3.3. Results of Clinical Studies

Autologous bone-marrow-derived stem cells effects when injected in the subchondral bone were evaluated in three studies: two randomized studies (Table 2) compared the results to total knee arthroplasty in the contralateral knee (one in young adults, the other on older population). Another study compared subchondral and intra-articular injection in the same patients.

- In a prospective randomized controlled clinical trial [16] carried out in 60 knees of 30 young patients who presented bilateral OA, one knee received a total knee arthroplasty (TKA) and the other knee received a subchondral bone marrow graft. At a follow up of average of 12 years (range 8 to 16 years), six (out of 30) TKA knees needed subsequent surgery versus only 1 with cell therapy. Knees with cell therapy had improvement on cartilage and bone marrow lesions observed at the site of bone marrow subchondral injection.
- In another prospective study, Hernigou et al. [45] included 140 adults aged 65 to 90 years. These 140 patients (mean age 75.4 ± 14.2 years) planned to undergo staged-bilateral total knee arthroplasty (TKA) for medial osteoarthritis, had “comparable” pain in both knees, and accepted randomization of the knees for surgery. They received TKA on one side and a subchondral injection of MSCs (from iliac bone marrow concentrate) on the contralateral knee during the same anesthetic. This study showed that subchondral bone marrow concentrate (as compared with TKA) had a sufficient effect on pain to postpone or avoid the TKA in the contralateral joint of patients with bilateral osteoarthritis.
- In a third randomized controlled clinical trial performed between 2000 and 2005, 60 patients with a similar osteoarthritis grade [26] received BMAC in the subchondral bone of one knee while the joint of the contralateral knee received intra-articular BMAC.

These three series represent 200 knees where the subchondral bone was injected for OA treatment in patients aged from 20 to 85 years. This BMAC graft represents (compared to the concentration in the subchondral bone before implantation) between a 60-fold increase and a 200-fold increase according to the severity of OA and age, with the higher increase for those knees with higher grade OA in younger patients. Improvement in VAS pain scores from baseline was observed for all knees (Kellgren–Lawrence grades 1 to 4) within each treatment group and for each follow-up time point until 15 years follow-up. Progression of joint space narrowing was not observed in some patients treated with subchondral MSCs. These results suggest that BM-MSCs injected in the subchondral bone may halt the progressive loss of cartilage observed in patients with OA. This was confirmed when cartilage volume changes over time were measured with MRI; the percentage cartilage volume measured with MRI (excluding osteophytes) on the medial compartment increased compared to baseline (2.4% ± 1.6% at 2 years). However, when revising for failures, only fibrocartilage was observed on histology. After treatment with BMAC injection in the subchondral bone, medial femorotibial compartment bone marrow lesions (BMLs) volume experienced regression over 24 months (mean regression 2.1 cm^3^, range 1.4 to 2.9 cm^3^) in all the knees. At 24 months, the knees with subchondral BMAC implantation demonstrated a 25% regression of the synovitis scores as compared to baseline, and a decrease of progenitors was observed in the synovial fluid of patients when aspiration of the joint was performed at 2 years follow-up. With a follow-up of average 13 years (range, 9 to 16 years) among the 200 knees treated with subchondral cell therapy, only 39 knees (20%) underwent TKA, suggesting the possibility to delay joint replacement through the application of a regenerative therapy.

### 3.4. Proposed Mechanism of Action BMAC Injected in the Subchondral Bone

**Figure 5:**
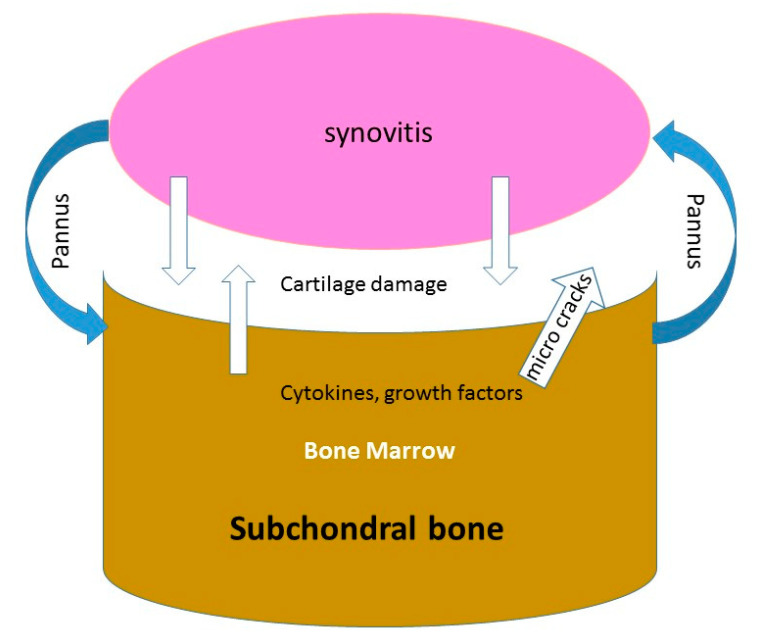
Schematic illustration of the interaction between synovitis, cartilage and subchondral bone in OA.

As synovitis decreased after subchondral injection of progenitors, some questions are as follows: How may subchondral injection decrease synovitis? How can MSCs injected in the subchondral bone decrease the number of SF MSCs in the fluid joint? A local interaction between subchondral bone-marrow tissue and synovial fluid exists in osteoarthritis as in rheumatoid inflammatory arthritis. Both marrow-to-synovium and synovium-to-marrow theories have been proposed for the pathogenesis [46]. The demonstration of channels between the subchondral bone and the uncalcified cartilage [47,48,49,50] and the presence of microcracks with vascularization in the subchondral bone plate might facilitate the transfer of benefit cytokines from the subchondral bone to the cartilage (Figure 5). Some penetrations of the cartilage and subchondral bone by a “pannus-like” tissue exist in the femorotibial compartment in OA, but this penetration causes much fewer erosions than those observed in rheumatoid arthritis. The reduction in the number of MSCs in the synovial fluid as the reduction of synovitis after MSCs subchondral injection could be a consequence of signals transmitted from the subchondral bone to the joint through the channels and vessels (or through the pannus) breaching the osteochondral-synovitis junction [51]. Other authors have reported with PRP injections better outcome with subchondral injections when compared with knee OA intra-articular injections alone [52,53,54,55]. Sanchez et al. [56] also suggested the possibility to delay TKA with subchondral PRP injections.

## 4. Scaffolds Loaded with BMAC as Beginning of Cartilage Engineering

### 4.1. The Rationale to Remove the Subchondral Bone in Osteoarthritis

Bone resorption pits in the subchondral bone may be a cause of cartilage damage, via the release of proteases, with a link between altered metabolism in subchondral bone and cartilage loss. Two enzymes produced in the subchondral bone (resorption areas), cathepsin K and MMP-13, may contribute to the degradation of macromolecules situated in the articular cartilage matrix. Another factor found in subchondral bone (TGF-β) could be a factor for cartilage degradation. The level of TGF- β is increased in the deep zone of OA cartilage, and in OA subchondral bone osteoblasts [57,58,59]. This distribution of TGF- β associated with its receptors in cartilage suggests that TGF-β could upregulate MMP-13 in OA cartilage. 

### 4.2. Scaffolds: Towards Biocartilage

Currently, the most innovative new treatment for osteochondral lesions involves tissue engineering, which consists of seeding a biomaterial with progenitor cells. The biomaterial must be biocompatible with the normal cartilage located in the surrounding site. It thus serves as scaffold for the synthesis of a new reparative tissue. The functionality of the newly formed tissue is determined by the endogenous presence of the growth factor at the lesional site, and by the mechanical properties of the new biomaterial to resist the forces of compression imposed by the movement of the articulation. 

The ideal scaffold should be nontoxic, biofunctional, biocompatible, biodegradable and easy to use. The inclusion of BMAC in a three-dimensional scaffold should provide an environment that will not only promote cellular differentiation but will also maintain the cells in the lesion. Different supports of synthetic, organic or even hybrid origin have been proposed. Examples of synthetic components include polylactic and polyglycolic acid. Supports of organic origin are composed of fibrin and of collagen fibers, with the collagen fibers being used either in the form of hydrogels or in the form of a sponge. Research has shown that collagen sponges are superior to collagen hydrogels in promoting the proliferation of cells as the synthesis of collagen and proteoglycans. From a mechanical point of view, the hydrogels present the advantage of using water in the same way as cartilage [60]. Under the force of compression, water pushed out of the hydrogel, which allows the latter to absorb the shock. Then, once the compressive force is released, the water is returned back to its place in the hydrogel, thus allowing it to return to its initial volume. From a biological point of view, the hydrogels provide a three-dimensional environment that is sufficiently porous to allow the proliferation of cells as well as the transportation of nutrients. Among the hydrogels, those containing sodium alginate constitute the model of reference not only in terms of studies of cellular morphology and the synthesis of collagen and proteoglycans [61], but also in terms of mechanobiology [62,63]. Although sodium alginate is not a natural component of the extracellular matrix, it has a structure similar to that of the glycosaminoglycan of cartilage. It has also been shown that this type of hydrogel ensures the maintenance of chondrocytes phenotypes [64]. 

### 4.3. Rationale to Load Scaffolds with BMAC

Several types of cells have been described for their potential application in the engineering of cartilage tissue such as mature cells (chondrocytes) or immature cells (mesenchymal stem ceIls). The utilization of each type of cell has its advantages and its disadvantages due to intrinsic biological properties. For example, the advantage of using BMAC with MSCs rather than autologous chondrocytes in the scaffold to repair cartilage is that chondrocytes can present the problem of their dedifferentiation during the course of their amplification in vivo. Recent studies have proposed cells that are less mature such as the mesenchymal stem cells. Gigante et al. [65] were the first to report treatment of symptomatic condylar defects of the knee. Five patients had a collagen type I scaffold implanted with arthroscopy. The scaffold was seeded with BMAC to induced chondrogenesis. Patients consented to a second-look arthroscopy at 12 months follow-up; histological examination revealed hyaline-like matrix in one case, a mixture of hyaline/fibrocartilage in one case and fibrocartilage in the rest of the cases.

### 4.4. Clinical Results in Long Term

Skowronski et al. [66] reported favorable clinical outcomes of BMAC (Table 3) with collagen membranes in large chondral lesions. A group of 21 patients was treated with bone marrow concentrate. The analysis of MRI showed satisfactory reconstruction of the cartilaginous surface and good regenerate integration. Gobbi et al. [67] followed prospectively (for a minimum of 6 years) twenty-three patients treated with HA (hyaluronic acid)-BMAC. Gobbi observed hyaline like cartilage in 80% of patients on magnetic resonance (MRI) imaging. Comparative analysis of pre- and post-operative scores was performed. All scores were significantly increased at the final follow-up. Buda et al. [68] reported 20 patients with osteochondral lesions treated with BMAC and scaffold. Histology showed cartilaginous tissue containing predominantly type II and proteoglycan-rich matrix. 

In summary, the technics of scaffolds associated with BMAC make this option an attractive possibility for cartilage restoration given the one-stage nature of the procedure and the long-term clinical benefits reflected by the current findings on large surfaces of defect. The treatment results may be viewed as satisfactory, as there were over 80% good and excellent results. The results are 10–15% inferior to those seen with 1st and 2nd generation autologous chondrocyte transplantation [69]. However, the costs associated with HA-BMAC make this an attractive option for cartilage restoration. Apart from the use of a commercial matrix and an available system to concentrate BMAC, the costs are similar to other widely used methods. This is in contrast to expanded in vitro chondrocytes which requires very expansive processing costs [70,71].

### 4.5. Proposed Mechanism of Scaffolds Loaded with BMAC to Produce Hyaline Cartilage

It is difficult to consider a new medicinal product without understanding the molecular mechanism of action. Particularly, it is difficult to understand why a low number of MSCs loaded in a scaffold can provide a similar result as a high number of expanded cells to regenerate cartilage. One of the obstacles for the development of MSC and BMAC therapy was the absence of specific mechanistic information. This may change because innovative studies have pushed research in unanticipated directions. Some intriguing new concepts have emerged to rationalize clinical effectiveness: secretome [72], apoptosis [73,74], and extracellular “vesicles” (EVs) [75]. A frequent observation in studies tracking delivered cells is the low efficiency of engraftment. It appears that MSCs show very low levels (3% or less) of engraftment with a rapid clearance, but still with positive effect. The MSC “secretome” or secreted proteins is probably one answer. MSCs, when delivered, provide to the host these factors which in turn indicates a repair response [72]. In a recent study, de Witte et al. [74] showed that the phagocytosis of transplanted MSCs by the monocytes enable monocytes of the host to mediate the immunomodulatory response after “MSC infusion”. This may explain persistence of a therapeutic response following MSC, despite their rapid clearance, and it may suggest that the long-term repair of a scaffold just loaded with BMAC containing a low number of MSCs is possible with a transient engraftment. There are probably many differences in protein secretion between the “in vitro MSC” and the “transplanted cell”. The MSC becomes a “responder cell” after transplantation and is just “licensed by the host” to provide molecules that may not be expressed in culture. This means that an approach may work in vivo even if this approach seems less favorable in vitro. Some report [75] has indicated that the responses observed following MSC transplantation can be obtained by the EV fraction alone. The idea that MSCs are only restorative and regenerating new tissue is insufficient; their action is probably more related to the host characteristics.

## 5. Discussion: Balancing the Different Methods of Treatment

### 5.1. Merits of and Demerits of Intra-Articular BMACs Injections

The major advantage of intra-articular injection is its convenience; it does not require general anesthesia and can be done under local anesthesia; an experienced physician can perform it without fluoroscopy; its downside is the short duration of efficiency reported by all series with potential for improvement of around one year.

### 5.2. Merits of and Demerits of Subchondral BMACs Injections

The use of subchondral injections as such does not have major demerits; however, if the injection can be carried out percutaneously as for the intra-articular injection, the technical level is more demanding; it requires the use of a fluoroscopy device, and the femoral condyle requires a physician with experience of triangulation to accurately place the trocar of injection under fluoroscopy (utilizing multiplanar images) to avoid any entry into the cartilage. Finally, if it is possible to perform it under local anesthesia, it may require, depending on the patient’s apprehension, greater analgesia or even general anesthesia. Its main advantage is the long-term improvement effect, lasting several years after injection.

### 5.3. Merits of and Demerits of Scaffolds Loaded with BMACs

The use of a scaffold has the drawbacks of being an open surgery and therefore a more invasive technique for the patient; it carries the usual risks of knee surgery, i.e., phlebitis, pulmonary embolism and infection. It requires the prescription of anticoagulant post-operatively. However, with this technique, the advantage is that scaffolds are capable of withstanding the loading environment and protect the loaded cells from any mechanical stresses. The new scaffolds can be considered as composite and bioactive materials for tissue engineering.

The main factors in favor or against each technique are reported in Table 4. Keeping in mind the advantages and disadvantages of each technique, it is worthwhile recalling that other alternatives may be considered for monitoring these patients, such as, for example, mesenchymal stem cells obtained from other tissues [3]. The decision between the different strategies should be based on the discussion with the patient, the personal experience of each physician and the availability and access to the different therapeutic facilities.

## 6. Conclusions

This review analyzed different technics with BMAC to treat osteoarthritic knees. Some studies had a small size of knees available, while other studies were retrospective studies. Another significant limitation is the heterogeneous characteristics of the control groups (exercise, saline, PRP, adipose tissue or TKA…). Moreover, BMAC application modalities (cells count, injected amount, injection schedule) were often different or not reported. This is probably a consequence of the young and recent scientific area of MSC therapy, in which there is no consensus about the ideal dose and preparation of cells. The optimal number of MSCs to treat knee OA was not the topic of this research. It is well known that the number of BM-MSCs present in the bone marrow of the iliac crest decreased with age, and this may be a limit because OA occurs rather in elderly patients. However, the findings of this review would suggest that the simple implantation of BMAC in an osteoarthritic knee could improve pain; the survivorship of the joint and could delay the need for arthroplasty.

## Figures and Tables

**Table 2:** Clinical randomized studies with BMAC subchondral injections.

Author	Study Design	Comparison	Nb of Knees	Age	Follow-Up	MRI
Hernigou [16]	Unblinded RCT	BMAC vs TKA	30/30	28 (18–41)	12 years	Yes
Hernigou [45]	Unblinded RCT	BMAC vs TKA	140/140	75(65–90)	15 years	Yes

**Table 3:** Clinical studies with BMAC loaded in scaffolds.

Author	Study Design	Scaffold	Nb of Knees	Age	Follow-Up	Biopsy/MRI
Gigante [65]	Retrospective	Collagen	5	43(25–54)	1 year	second look biopsies
Skowronski [66]	Retrospective	Collagen	21	26(17–52)	5 years	MRI
Gobi [67]	Prospective	Hyaluronic Acid	23	48 ± 9	8 years	MRI
Buda [68]	Retrospective	Hyaluronic Acid	20	35(15–50)	2 years	Biopsies

**Table 4:** Pros and Cons of different arguments in BMAC administration.

Technique	In Favor	Against
Intra-articular	percutaneous	short term efficiency
	Local anesthesia	
Subchondral	percutaneous	needs fluoroscopy
	Long term efficiency	±general anesthesia
Scaffolds	protect cells	open surgery
	Bioactive material	potential complications

## Data Availability

Not applicable.

## References

[1] Caplan A.I. (2005). Review: Mesenchymal Stem Cells: Cell-Based Reconstructive Therapy in Orthopedics. Tissue Eng..

[2] Chen F.H., Tuan R.S. (2008). Mesenchymal Stem Cells in Arthritic Diseases. Arthritis Res. Ther..

[3] Kuçi S., Henschler R., Müller I., Biagi E., Meisel R. (2012). Basic Biology and Clinical Application of Multipotent Mesenchymal Stromal Cells: From Bench to Bedside. Stem Cells Int..

[4] Friedenstein A.J., Piatetzky-Shapiro I.I., Petrakova K.V. (1966). Osteogenesis in Transplants of Bone Marrow Cells. J. Embryol. Exp. Morphol..

[5] Pagani S., Borsari V., Veronesi F., Ferrari A., Cepollaro S., Torricelli P., Filardo G., Fini M. (2017). Increased Chondrogenic Potential of Mesenchymal Cells From Adipose Tissue Versus Bone Marrow-Derived Cells in Osteoarthritic In Vitro Models. J. Cell Physiol..

[6] Turner L., Knoepfler P. (2016). Selling Stem Cells in the USA: Assessing the Direct-to-Consumer Industry. Cell Stem Cell.

[7] Song L., Tuan R.S. (2004). Transdifferentiation Potential of Human Mesenchymal Stem Cells Derived from Bone Marrow. FASEB J..

[8] Herberts C.A., Kwa M.S.G., Hermsen H.P.H. (2011). Risk Factors in the Development of Stem Cell Therapy. J. Transl. Med..

[9] Desando G., Bartolotti I., Cavallo C., Schiavinato A., Secchieri C., Kon E., Filardo G., Paro M., Grigolo B. (2018). Short-Term Homing of Hyaluronan-Primed Cells: Therapeutic Implications for Osteoarthritis Treatment. Tissue Eng. Part C Methods.

[10] Wang Z., Zhai C., Fei H., Hu J., Cui W., Wang Z., Li Z., Fan W. (2018). Intraarticular Injection Autologous Platelet-Rich Plasma and Bone Marrow Concentrate in a Goat Osteoarthritis Model. J. Orthop. Res..

[11] Song F., Tang J., Geng R., Hu H., Zhu C., Cui W., Fan W. (2014). Comparison of the Efficacy of Bone Marrow Mononuclear Cells and Bone Mesenchymal Stem Cells in the Treatment of Osteoarthritis in a Sheep Model. Int. J. Clin. Exp. Pathol..

[12] Singh A., Goel S.C., Gupta K.K., Kumar M., Arun G.R., Patil H., Kumaraswamy V., Jha S. (2014). The Role of Stem Cells in Osteoarthritis: An Experimental Study in Rabbits. Bone Joint Res..

[13] Hernigou J., Vertongen P., Chahidi E., Kyriakidis T., Dehoux J.-P., Crutzen M., Boutry S., Larbanoix L., Houben S., Gaspard N. (2018). Effects of Press-Fit Biphasic (Collagen and HA/ΒTCP) Scaffold with Cell-Based Therapy on Cartilage and Subchondral Bone Repair Knee Defect in Rabbits. Int. Orthop..

[14] Filardo G., Perdisa F., Roffi A., Marcacci M., Kon E. (2016). Stem Cells in Articular Cartilage Regeneration. J. Orthop. Surg. Res..

[15] Gobbi A., Karnatzikos G., Scotti C., Mahajan V., Mazzucco L., Grigolo B. (2011). One-Step Cartilage Repair with Bone Marrow Aspirate Concentrated Cells and Collagen Matrix in Full-Thickness Knee Cartilage Lesions: Results at 2-Year Follow-Up. Cartilage.

[16] Hernigou P., Auregan J.C., Dubory A., Flouzat-Lachaniette C.H., Chevallier N., Rouard H. (2018). Subchondral Stem Cell Therapy versus Contralateral Total Knee Arthroplasty for Osteoarthritis Following Secondary Osteonecrosis of the Knee. Int. Orthop..

[17] Yusup A., Kaneko H., Liu L., Ning L., Sadatsuki R., Hada S., Kamagata K., Kinoshita M., Futami I., Shimura Y. (2015). Bone Marrow Lesions, Subchondral Bone Cysts and Subchondral Bone Attrition Are Associated with Histological Synovitis in Patients with End-Stage Knee Osteoarthritis: A Cross-Sectional Study. Osteoarthr. Cartil..

[18] Egloff C., Hügle T., Valderrabano V. (2012). Biomechanics and Pathomechanisms of Osteoarthritis. Swiss Med. Wkly..

[19] Atukorala I., Kwoh C.K., Guermazi A., Roemer F.W., Boudreau R.M., Hannon M.J., Hunter D.J. (2016). Synovitis in Knee Osteoarthritis: A Precursor of Disease?. Ann. Rheum. Dis..

[20] Schueller-Weidekamm C., Schueller G., Uffmann M., Bader T.R. (2006). Does Marathon Running Cause Acute Lesions of the Knee? Evaluation with Magnetic Resonance Imaging. Eur. Radiol..

[21] Loeuille D., Chary-Valckenaere I., Champigneulle J., Rat A.-C., Toussaint F., Pinzano-Watrin A., Goebel J.C., Mainard D., Blum A., Pourel J. (2005). Macroscopic and Microscopic Features of Synovial Membrane Inflammation in the Osteoarthritic Knee: Correlating Magnetic Resonance Imaging Findings with Disease Severity. Arthritis Rheum..

[22] Roemer F.W., Kassim Javaid M., Guermazi A., Thomas M., Kiran A., Keen R., King L., Arden N.K. (2010). Anatomical Distribution of Synovitis in Knee Osteoarthritis and Its Association with Joint Effusion Assessed on Non-Enhanced and Contrast-Enhanced MRI. Osteoarthr. Cartil..

[23] Jones E.A., Crawford A., English A., Henshaw K., Mundy J., Corscadden D., Chapman T., Emery P., Hatton P., McGonagle D. (2008). Synovial Fluid Mesenchymal Stem Cells in Health and Early Osteoarthritis: Detection and Functional Evaluation at the Single-Cell Level. Arthritis Rheum..

[24] Sekiya I., Ojima M., Suzuki S., Yamaga M., Horie M., Koga H., Tsuji K., Miyaguchi K., Ogishima S., Tanaka H. (2012). Human Mesenchymal Stem Cells in Synovial Fluid Increase in the Knee with Degenerated Cartilage and Osteoarthritis. J. Orthop. Res..

[25] Davies B.M., Snelling S.J.B., Quek L., Hakimi O., Ye H., Carr A., Price A.J. (2017). Identifying the Optimum Source of Mesenchymal Stem Cells for Use in Knee Surgery. J. Orthop. Res..

[26] Hernigou P., Bouthors C., Bastard C., Flouzat Lachaniette C.H., Rouard H., Dubory A. (2021). Subchondral Bone or Intra-Articular Injection of Bone Marrow Concentrate Mesenchymal Stem Cells in Bilateral Knee Osteoarthritis: What Better Postpone Knee Arthroplasty at Fifteen Years? A Randomized Study. Int. Orthop..

[27] Kellgren J.H., Lawrence J.S. (1957). Radiological Assessment of Osteo-Arthrosis. Ann. Rheum. Dis..

[28] Mautner K., Bowers R., Easley K., Fausel Z., Robinson R. (2019). Functional Outcomes Following Microfragmented Adipose Tissue Versus Bone Marrow Aspirate Concentrate Injections for Symptomatic Knee Osteoarthritis. Stem Cells Transl. Med..

[29] Shaw B., Darrow M., Derian A. (2018). Short-Term Outcomes in Treatment of Knee Osteoarthritis With 4 Bone Marrow Concentrate Injections. Clin. Med. Insights Arthritis Musculoskelet. Disord..

[30] Themistocleous G.S., Chloros G.D., Kyrantzoulis I.M., Georgokostas I.A., Themistocleous M.S., Papagelopoulos P.J., Savvidou O.D. (2018). Effectiveness of a Single Intra-Articular Bone Marrow Aspirate Concentrate (BMAC) Injection in Patients with Grade 3 and 4 Knee Osteoarthritis. Heliyon.

[31] Shapiro S.A., Kazmerchak S.E., Heckman M.G., Zubair A.C., O’Connor M.I. (2017). A Prospective, Single-Blind, Placebo-Controlled Trial of Bone Marrow Aspirate Concentrate for Knee Osteoarthritis. Am. J. Sports Med..

[32] Centeno C., Sheinkop M., Dodson E., Stemper I., Williams C., Hyzy M., Ichim T., Freeman M. (2018). A Specific Protocol of Autologous Bone Marrow Concentrate and Platelet Products versus Exercise Therapy for Symptomatic Knee Osteoarthritis: A Randomized Controlled Trial with 2 Year Follow-Up. J. Transl. Med..

[33] Goncars V., Jakobsons E., Blums K., Briede I., Patetko L., Erglis K., Erglis M., Kalnberzs K., Muiznieks I., Erglis A. (2017). The Comparison of Knee Osteoarthritis Treatment with Single-Dose Bone Marrow-Derived Mononuclear Cells vs. Hyaluronic Acid Injections. Medicina.

[34] Anz A.W., Hubbard R., Rendos N.K., Everts P.A., Andrews J.R., Hackel J.G. (2020). Bone Marrow Aspirate Concentrate Is Equivalent to Platelet-Rich Plasma for the Treatment of Knee Osteoarthritis at 1 Year: A Prospective, Randomized Trial. Orthop. J. Sports Med..

[35] Montaseri A., Busch F., Mobasheri A., Buhrmann C., Aldinger C., Rad J.S., Shakibaei M. (2011). IGF-1 and PDGF-Bb Suppress IL-1β-Induced Cartilage Degradation through down-Regulation of NF-ΚB Signaling: Involvement of Src/PI-3K/AKT Pathway. PLoS ONE.

[36] Richardson D., Pearson R.G., Kurian N., Latif M.L., Garle M.J., Barrett D.A., Kendall D.A., Scammell B.E., Reeve A.J., Chapman V. (2008). Characterisation of the Cannabinoid Receptor System in Synovial Tissue and Fluid in Patients with Osteoarthritis and Rheumatoid Arthritis. Arthritis Res. Ther..

[37] Hayashi D., Englund M., Roemer F.W., Niu J., Sharma L., Felson D.T., Crema M.D., Marra M.D., Segal N.A., Lewis C.E. (2012). Knee Malalignment Is Associated with an Increased Risk for Incident and Enlarging Bone Marrow Lesions in the More Loaded Compartments: The MOST Study. Osteoarthr. Cartil..

[38] Englund M., Guermazi A., Roemer F.W., Yang M., Zhang Y., Nevitt M.C., Lynch J.A., Lewis C.E., Torner J., Felson D.T. (2010). Meniscal Pathology on MRI Increases the Risk for Both Incident and Enlarging Subchondral Bone Marrow Lesions of the Knee: The MOST Study. Ann. Rheum. Dis..

[39] Hunter D.J., Zhang Y., Niu J., Goggins J., Amin S., LaValley M.P., Guermazi A., Genant H., Gale D., Felson D.T. (2006). Increase in Bone Marrow Lesions Associated with Cartilage Loss: A Longitudinal Magnetic Resonance Imaging Study of Knee Osteoarthritis. Arthritis Rheum..

[40] Lisignoli G., Toneguzzi S., Piacentini A., Cristino S., Grassi F., Cavallo C., Facchini A. (2006). CXCL12 (SDF-1) and CXCL13 (BCA-1) Chemokines Significantly Induce Proliferation and Collagen Type I Expression in Osteoblasts from Osteoarthritis Patients. J. Cell Physiol..

[41] Massicotte F., Lajeunesse D., Benderdour M., Pelletier J.-P., Hilal G., Duval N., Martel-Pelletier J. (2002). Can Altered Production of Interleukin-1beta, Interleukin-6, Transforming Growth Factor-Beta and Prostaglandin E(2) by Isolated Human Subchondral Osteoblasts Identify Two Subgroups of Osteoarthritic Patients. Osteoarthr. Cartil..

[42] Paredes Y., Massicotte F., Pelletier J.-P., Martel-Pelletier J., Laufer S., Lajeunesse D. (2002). Study of the Role of Leukotriene B()4 in Abnormal Function of Human Subchondral Osteoarthritis Osteoblasts: Effects of Cyclooxygenase and/or 5-Lipoxygenase Inhibition. Arthritis Rheum..

[43] Hilal G., Martel-Pelletier J., Pelletier J.P., Ranger P., Lajeunesse D. (1998). Osteoblast-like Cells from Human Subchondral Osteoarthritic Bone Demonstrate an Altered Phenotype in Vitro: Possible Role in Subchondral Bone Sclerosis. Arthritis Rheum..

[44] Aspden R.M., Scheven B.A., Hutchison J.D. (2001). Osteoarthritis as a Systemic Disorder Including Stromal Cell Differentiation and Lipid Metabolism. Lancet.

[45] Hernigou P., Delambre J., Quiennec S., Poignard A. (2021). Human Bone Marrow Mesenchymal Stem Cell Injection in Subchondral Lesions of Knee Osteoarthritis: A Prospective Randomized Study versus Contralateral Arthroplasty at a Mean Fifteen Year Follow-Up. Int. Orthop..

[46] Hügle T., Geurts J. (2017). What Drives Osteoarthritis?-Synovial versus Subchondral Bone Pathology. Rheumatology.

[47] Burr D.B., Radin E.L. (2003). Microfractures and Microcracks in Subchondral Bone: Are They Relevant to Osteoarthrosis?. Rheum. Dis. Clin. North. Am..

[48] Villanueva A.R., Longo J.A., Weiner G. (1994). Staining and Histomorphometry of Microcracks in the Human Femoral Head. Biotech. Histochem..

[49] Sokoloff L. (1993). Microcracks in the Calcified Layer of Articular Cartilage. Arch. Pathol. Lab. Med..

[50] Clark J.M. (1990). The Structure of Vascular Channels in the Subchondral Plate. J. Anat..

[51] Endres M., Neumann K., Häupl T., Erggelet C., Ringe J., Sittinger M., Kaps C. (2007). Synovial Fluid Recruits Human Mesenchymal Progenitors from Subchondral Spongious Bone Marrow. J. Orthop. Res..

[52] Lychagin A., Lipina M., Garkavi A., Islaieh O., Timashev P., Ashmore K., Kon E. (2021). Intraosseous Injections of Platelet Rich Plasma for Knee Bone Marrow Lesions Treatment: One Year Follow-Up. Int. Orthop..

[53] Su K., Bai Y., Wang J., Zhang H., Liu H., Ma S. (2018). Comparison of Hyaluronic Acid and PRP Intra-Articular Injection with Combined Intra-Articular and Intraosseous PRP Injections to Treat Patients with Knee Osteoarthritis. Clin. Rheumatol..

[54] Sánchez M., Delgado D., Sánchez P., Muiños-López E., Paiva B., Granero-Moltó F., Prósper F., Pompei O., Pérez J.C., Azofra J. (2016). Combination of Intra-Articular and Intraosseous Injections of Platelet Rich Plasma for Severe Knee Osteoarthritis: A Pilot Study. Biomed. Res. Int..

[55] Sánchez M., Delgado D., Pompei O., Pérez J.C., Sánchez P., Garate A., Bilbao A.M., Fiz N., Padilla S. (2019). Treating Severe Knee Osteoarthritis with Combination of Intra-Osseous and Intra-Articular Infiltrations of Platelet-Rich Plasma: An Observational Study. Cartilage.

[56] Sánchez M., Fiz N., Guadilla J., Padilla S., Anitua E., Sánchez P., Delgado D. (2014). Intraosseous Infiltration of Platelet-Rich Plasma for Severe Knee Osteoarthritis. Arthrosc. Tech..

[57] Fernandes J.C., Martel-Pelletier J., Lascau-Coman V., Moldovan F., Jovanovic D., Raynauld J.P., Pelletier J.P. (1998). Collagenase-1 and Collagenase-3 Synthesis in Normal and Early Experimental Osteoarthritic Canine Cartilage: An Immunohistochemical Study. J. Rheumatol..

[58] Moldovan F., Pelletier J.P., Hambor J., Cloutier J.M., Martel-Pelletier J. (1997). Collagenase-3 (Matrix Metalloprotease 13) Is Preferentially Localized in the Deep Layer of Human Arthritic Cartilage in Situ: In Vitro Mimicking Effect by Transforming Growth Factor Beta. Arthritis Rheum..

[59] Moldovan F., Pelletier J.P., Mineau F., Dupuis M., Cloutier J.M., Martel-Pelletier J. (2000). Modulation of Collagenase 3 in Human Osteoarthritic Cartilage by Activation of Extracellular Transforming Growth Factor Beta: Role of Furin Convertase. Arthritis Rheum..

[60] Häuselmann H.J., Aydelotte M.B., Schumacher B.L., Kuettner K.E., Gitelis S.H., Thonar E.J. (1992). Synthesis and Turnover of Proteoglycans by Human and Bovine Adult Articular Chondrocytes Cultured in Alginate Beads. Matrix.

[61] Wong M., Siegrist M., Wang X., Hunziker E. (2001). Development of Mechanically Stable Alginate/Chondrocyte Constructs: Effects of Guluronic Acid Content and Matrix Synthesis. J. Orthop. Res..

[62] Ragan P.M., Chin V.I., Hung H.H., Masuda K., Thonar E.J., Arner E.C., Grodzinsky A.J., Sandy J.D. (2000). Chondrocyte Extracellular Matrix Synthesis and Turnover Are Influenced by Static Compression in a New Alginate Disk Culture System. Arch. Biochem. Biophys..

[63] Gigant-Huselstein C., Dumas D., Hubert P., Baptiste D., Dellacherie E., Mainard D., Netter P., Payan E., Stoltz J.F. (2003). Influence of Mechanical Stress on Extracellular Matrixes Synthesized by Chondrocytes Seeded onto Alginate and Hyaluronate-Based 3d Biosystems. J. Mech. Med. Biol..

[64] Miralles G., Baudoin R., Dumas D., Baptiste D., Hubert P., Stoltz J.F., Dellacherie E., Mainard D., Netter P., Payan E. (2001). Sodium Alginate Sponges with or without Sodium Hyaluronate: In Vitro Engineering of Cartilage. J. Biomed. Mater. Res..

[65] Gigante A., Calcagno S., Cecconi S., Ramazzotti D., Manzotti S., Enea D. (2011). Use of Collagen Scaffold and Autologous Bone Marrow Concentrate as a One-Step Cartilage Repair in the Knee: Histological Results of Second-Look Biopsies at 1 Year Follow-Up. Int. J. Immunopathol. Pharmacol..

[66] Skowroński J., Rutka M. (2013). Osteochondral Lesions of the Knee Reconstructed with Mesenchymal Stem Cells-Results. Ortop Traumatol. Rehabil..

[67] Gobbi A., Whyte G.P. (2019). Long-Term Clinical Outcomes of One-Stage Cartilage Repair in the Knee With Hyaluronic Acid-Based Scaffold Embedded With Mesenchymal Stem Cells Sourced From Bone Marrow Aspirate Concentrate. Am. J. Sports Med..

[68] Buda R., Vannini F., Cavallo M., Baldassarri M., Luciani D., Mazzotti A., Pungetti C., Olivieri A., Giannini S. (2013). One-Step Arthroscopic Technique for the Treatment of Osteochondral Lesions of the Knee with Bone-Marrow-Derived Cells: Three Years Results. Musculoskelet. Surg..

[69] Brittberg M., Lindahl A., Nilsson A., Ohlsson C., Isaksson O., Peterson L. (1994). Treatment of Deep Cartilage Defects in the Knee with Autologous Chondrocyte Transplantation. N. Engl. J. Med..

[70] Peterson L., Vasiliadis H.S., Brittberg M., Lindahl A. (2010). Autologous Chondrocyte Implantation: A Long-Term Follow-Up. Am. J. Sports Med..

[71] Samuelson E.M., Brown D.E. (2012). Cost-Effectiveness Analysis of Autologous Chondrocyte Implantation: A Comparison of Periosteal Patch versus Type I/III Collagen Membrane. Am. J. Sports Med..

[72] Mancuso P., Raman S., Glynn A., Barry F., Murphy J.M. (2019). Mesenchymal Stem Cell Therapy for Osteoarthritis: The Critical Role of the Cell Secretome. Front. Bioeng. Biotechnol..

[73] Galleu A., Riffo-Vasquez Y., Trento C., Lomas C., Dolcetti L., Cheung T.S., von Bonin M., Barbieri L., Halai K., Ward S. (2017). Apoptosis in Mesenchymal Stromal Cells Induces in Vivo Recipient-Mediated Immunomodulation. Sci. Transl. Med..

[74] De Witte S.F.H., Luk F., Sierra Parraga J.M., Gargesha M., Merino A., Korevaar S.S., Shankar A.S., O’Flynn L., Elliman S.J., Roy D. (2018). Immunomodulation By Therapeutic Mesenchymal Stromal Cells (MSC) Is Triggered Through Phagocytosis of MSC By Monocytic Cells. Stem Cells.

[75] Zhang S., Chuah S.J., Lai R.C., Hui J.H.P., Lim S.K., Toh W.S. (2018). MSC Exosomes Mediate Cartilage Repair by Enhancing Proliferation, Attenuating Apoptosis and Modulating Immune Reactivity. Biomaterials.

